# Chloroform Treatment in Typhoid Fever

**Published:** 1891-04

**Authors:** 

**Affiliations:** Nuremberg


### CHLOROFORM TREATMENT IN TYPHOID FEVER.

Dr. Hepp, Nuremberg.

1.0 : 150.0, divided into three doses, is the daily maximum which Hepp has now used for nearly two years in the treatment of typhoid, as well as in acute affections of the digestive tract, in chronic ulcer of the stomach, and in croupous pneumonia. Even in grave cases its influence soon shows itself, the somnolence and the deliria cease, the dry skin becomes moist and the patient feels better ; the temperature slowly but steadily falls, and the remittent stage soon leads to convalescence. Chloroform passes as such, without decomposing, through the body, and only thus its favorable, anti-bacterial action can be explained, and it must be considered as a stimulant. It is hardly possible that this small daily dose of 1.0 : 150.0 can ever do any harm in relation to dissolving the blood corpuscles, for where the disease may be prolonged for several weeks the quantity consumed would not be over twenty grammes, while during anæsthesia as much as 150.0 grammes were used during several hours.

Munich, M. W., 45 '90.

Allen, III, p. 263, gives the following symptoms, which strongly hint to the efficacy of Chloroform in typhoid and similar diseases, and show its homœopathicity to them. 8. Seemed scarcely to understand anything said to him, and kept on muttering; 15 and 16. Complete unconsciousness ; 74. Roaring in ears; 81. Cadaverous countenance or intoxicated expression of face (Baptisia); 91. Tongue dry and parchment like ; 92. Mouth half open ; 95. Could hardly articulate; 130. Abdomen tense ; 139 and 140. Involuntary defæcation, bloody diarrhœa; 149. Bladder distended and bedclothes stained by the urine which had escaped ; 158. Tracheal râles, stertorous respiration, irregular breathing ; 184. Moist crepitations through lungs ; 240. Relaxation of muscles, very feeble and still restless and tossing about, etc.

Hering, in his *Guiding Symptoms*, IV. 90, mentions clinically typhus fever (the German name for typhoid) with great prostration, delirium, subsultio, irregular respiration and nervous restlessness, but we think that the symptoms which he put down under Arachnitis really belong to these grave typhoid conditions, and, if given at an early stage, may prevent the fever from reaching its acme. Again, never mind the name of a disease, but be, whenever possible, a true healer. S. L.

